# Subcortical Structures in Humans Can Be Facilitated by Transcranial Direct Current Stimulation

**DOI:** 10.1371/journal.pone.0107731

**Published:** 2014-09-18

**Authors:** Jorik Nonnekes, Anass Arrogi, Moniek A. M. Munneke, Edwin H. F. van Asseldonk, Lars B. Oude Nijhuis, Alexander C. Geurts, Vivian Weerdesteyn

**Affiliations:** 1 Radboud University Medical Centre, Donders Institute for Brain, Cognition and Behaviour, Department of Rehabilitation, Nijmegen, The Netherlands; 2 Radboud University Medical Centre, Donders Institute for Brain, Cognition and Behaviour, Department of Neurology/Clinical Neurophysiology, Nijmegen, The Netherlands; 3 Department of Biomechanical Engineering, MIRA, University of Twente, Enschede, The Netherlands; 4 Sint Maartenskliniek Research, Development & Education, Nijmegen, The Netherlands; Purdue University, United States of America

## Abstract

Transcranial direct current stimulation (tDCS) is a noninvasive brain stimulation technique that alters cortical excitability. Interestingly, in recent animal studies facilitatory effects of tDCS have also been observed on subcortical structures. Here, we sought to provide evidence for the potential of tDCS to facilitate subcortical structures in humans as well. Subjects received anodal-tDCS and sham-tDCS on two separate testing days in a counterbalanced order. After stimulation, we assessed the effect of tDCS on two responses that arise from subcortical structures; (1) wrist and ankle responses to an imperative stimulus combined with a startling acoustic stimulus (SAS), and (2) automatic postural responses to external balance perturbations with and without a concurrent SAS. During all tasks, response onsets were significantly faster following anodal-tDCS compared to sham-tDCS, both in trials with and without a SAS. The effect of tDCS was similar for the dominant and non-dominant leg. The SAS accelerated the onsets of ankle and wrist movements and the responses to backward, but not forward perturbations. The faster onsets of SAS-induced wrist and ankle movements and automatic postural responses following stimulation provide strong evidence that, in humans, subcortical structures - in particular the reticular formation - can be facilitated by tDCS. This effect may be explained by two mechanisms that are not mutually exclusive. First, subcortical facilitation may have resulted from enhanced cortico-reticular drive. Second, the applied current may have directly stimulated the reticular formation. Strengthening reticulospinal output by tDCS may be of interest to neurorehabilitation, as there is evidence for reticulospinal compensation after corticospinal lesions.

## Introduction

Transcranial direct current stimulation (tDCS) is a noninvasive brain stimulation technique that alters cortical excitability via application of a weak direct current. The proposed neuronal mechanism underlying the observed facilitatory or inhibitory effects on cortical output involves slight shifts in the resting membrane potential of cortical neurons [1]–[3]. In humans, facilitation of cortical areas by means of anodal tDCS has been found to improve several motor and cognitive functions [4], [5], which has sparked a wealth of research on its utility in patients with central injuries. Interestingly, a recent study in anaesthetized cats showed that tDCS not only affects cortical excitability, but also facilitates subcortical neurons [6]. Rubrospinal and reticulospinal neurons were facilitated by anodal tDCS over the sensorimotor cortex, resulting in shortened latencies and/or increased amplitudes of descending volleys. Such remote effects of tDCS may greatly expand its potential utility in patients with central injuries, but whether tDCS can also facilitate subcortical structures in humans is yet unknown. An imaging study has suggested that subcortical facilitation by tDCS may indeed be possible in humans [7], but direct evidence is lacking. In this study, we sought to provide evidence for the potential of tDCS to facilitate subcortical structures in humans. We established the effect of tDCS in healthy human subjects on responses that originate from subcortical structures. First, we examined wrist and ankle responses to an imperative ‘go’ signal, both with and without simultaneous presentation of a startling acoustic stimulus (SAS). A SAS accelerates the onset latencies of movement responses [8], which has been termed ‘StartReact effect’. The shortened onset latencies reflect a direct subcortical release of motor programs [9]–[11]. Second, we examined automatic postural responses to external balance perturbations, with and without a concurrent SAS. These initial postural responses, both with and without a SAS, also arise from subcortical structures [12].

## Materials and Methods

### Participants

Ten healthy adults (4 women, mean 22 years, range 18–27) participated in this study. Nine participants were right-handed as verified by the Edinburgh Handedness Inventory [13]. These nine participants also showed dominance of the right leg, as identified by the question ‘with which foot would you kick a soccer ball’? None of the participants suffered from hearing, neurological or motor disorders that could interfere with performance during the experiments. The study was approved by the local medical ethics committee (CMO region Arnhem/Nijmegen) and was conducted in accordance with the Declaration of Helsinki. All subjects gave their written informed consent prior to the experiment.

### Experimental setup and protocol

Participants were measured on two different measurement sessions (separated by at least one week) in which they first received ‘anodal-tDCS’ or ‘sham-tDCS’. The order of the stimulation type was counterbalanced across subjects. Following stimulation, simple reaction times (wrist flexion and ankle dorsiflexion) and onset latencies of postural responses were evaluated. The order of the tasks was also counterbalanced across subjects.

We designed our protocol such that the assessments could be completed within 30 minutes after stimulation because of the time-limited effect of tDCS [4]. Due to these time limitations, we chose to assess wrist flexion unilaterally (ipsilateral to the hemisphere receiving anodal stimulation), in light of the evidence that arm flexors predominantly receive ipsilateral reticulospinal projections [14]. Ankle dorsiflexion was assessed bilaterally, as it is yet unknown whether reticulospinal projections to the dorsiflexor muscles are predominantly ipsilateral or contralateral.

#### tDCS

tDCS was applied by a battery-driven constant-current stimulator (DC-STIMULATOR PLUS, NeuroConn, Illmenau, Germany) via conductive-rubber electrodes, placed in two saline-soaked sponges (5×7 cm). The anodal electrode was placed over the non-dominant motor region (C3/C4 on the 10–20 international electroencephalogram system). The reference electrode was placed over the contralateral supraorbital region. We stimulated the non-dominant motor region, as we evaluated wrist flexion in the dominant arm, with the arm flexors receiving dominant ipsilateral cortico-reticular projections [14]. During anodal stimulation, tDCS was applied for 15 minutes at an intensity of 2 mA. The current was ramped up to its target intensity over 10 seconds and ramped down in the same time interval at the end of the stimulation period. During sham stimulation, the same procedure was followed but current was applied for 15 seconds only after the first ramp period, followed by 10 seconds ramp down. Stimulation was applied in standing position. Two participants were able to differentiate between the sham and anodal condition, whereas the remaining eight participants could not indicate which session involved anodal-tDCS.

#### Simple reaction time task

Participants sat in a chair with their hip, knee and ankle joints in 90 degrees. The chair was positioned 2.5 meters in front of two arrays of light-emitting diodes (LEDs; 11×8 cm, 3 cm apart). Illumination of the first LED array formed a warning signal. We instructed participants to respond as rapidly as possible to illumination of the second LED array (i.e., imperative stimulus) in three separate movement tasks; 1) dorsiflexion of the dominant or 2) non-dominant ankle, or 3) flexion of the dominant wrist. The order of the conditions was counterbalanced across subjects. Warning periods (1–3.5 seconds) and inter-trial periods (6–10 seconds) were variable. In each condition, participants performed 16 trials. In 25% of trials a startling acoustic stimulus (SAS) was given simultaneously with the imperative stimulus. The SAS was given through binaural earphones and consisted of 50 ms white noise with an intensity of 116 dB (sound pressure level), and was generated by a custom-made noise generator.

For the condition involving wrist flexion, the participant’s arm was secured in a semi-prone position with the palm facing inward to a custom-made wrist manipulandum that moved in the transverse plane with an axis of rotation at the wrist joint [11], [15].

#### Automatic postural responses

Participants stood on a moveable platform that could suddenly and unexpectedly translate in the forward or backward direction [16]. A forward translation of the platform resulted in a backward balance perturbation and vice versa. In the remainder of this text, we will refer to the direction of the balance perturbation. Platform movements comprised an acceleration phase (300 ms), a constant-velocity phase (500 ms) and a deceleration phase (300 ms). Both forward and backward perturbations were delivered by platform acceleration of 0.75 m/s^2^. Participants received 16 forward and 16 backward balance perturbations in a random order. In 25% of both forward and backward trials, the perturbation was combined with a SAS that was administered through binaural earphones at the start of the platform movement. Consecutive trials were separated by at least 20 seconds. On both testing days, subjects received four practice trials before tDCS (two for each direction). Participants were instructed to sustain the perturbations without taking a step or grabbing the handrails surrounding the platform for support.

### Data collection

Muscle activity was measured using surface electromyography (EMG) data from bilateral tibialis anterior and gastrocnemius medialis muscles and from the dominant flexor carpi radials muscle (ZeroWire by Aurion, Italy; 2000 Hz). Self-adhesive Ag-AgCl electrodes (Tyco Arbo ECG) were placed approximately 2 cm apart and longitudinally on the belly of each muscle, according to Seniam guidelines [17]. EMG signals were sampled at 2000 Hz and full-wave rectified and low-pass filtered at 30 Hz (zero-lag, second order Butterworth filter). Furthermore, to assess movement onset, a triaxial accelerometer was placed at the foot or hand involved in the simple reaction task. Accelerometer signals were sampled at 2000 Hz.

### Data analysis

#### Simple reaction time task

Two reaction time parameters were assessed: EMG reaction time and accelerometer reaction time. For each condition, we calculated ensemble average EMG and accelerometer traces, separately for trials with and without a SAS. Onset latencies of the muscles of interest were determined using a semi-automatic computer algorithm that selected the first instant at which the mean EMG activity exceeded a threshold of 2 standard deviations (SD) above the mean background activity, as calculated over a 500 ms period just prior to the imperative ‘go’ signal. Onsets were first selected by the computer algorithm, then visually approved and (when necessary) corrected [18]. Average onset latencies were calculated separately for trials with and without a SAS. The onset of foot and wrist acceleration was determined in the same manner.

#### Automatic postural responses

We determined the latencies of the prime movers of the postural responses using the algorithm described above. For forward perturbations, we identified the onset latencies in the gastrocnemius medialis muscle; for backward perturbations, we determined the onset latencies in the tibialis anterior muscle.

### Statistical analysis

Ankle dorsiflexion reaction times and latencies of automatic postural responses were evaluated using a repeated measures ANOVA, with SAS (*SAS – no SAS*), tDCS (*anodal-tDCS – sham-tDCS*) and leg (*dominant – non-dominant*) as within subjects factors. Wrist flexion reaction times were evaluated using SAS and tDCS as within subjects factors. Main effects are reported as well as *SAS x tDCS* effects. Other interaction effects are only reported if significant. The alpha level was set at 0.05.

## Results

### Simple reaction time tasks

**Figure 1:**
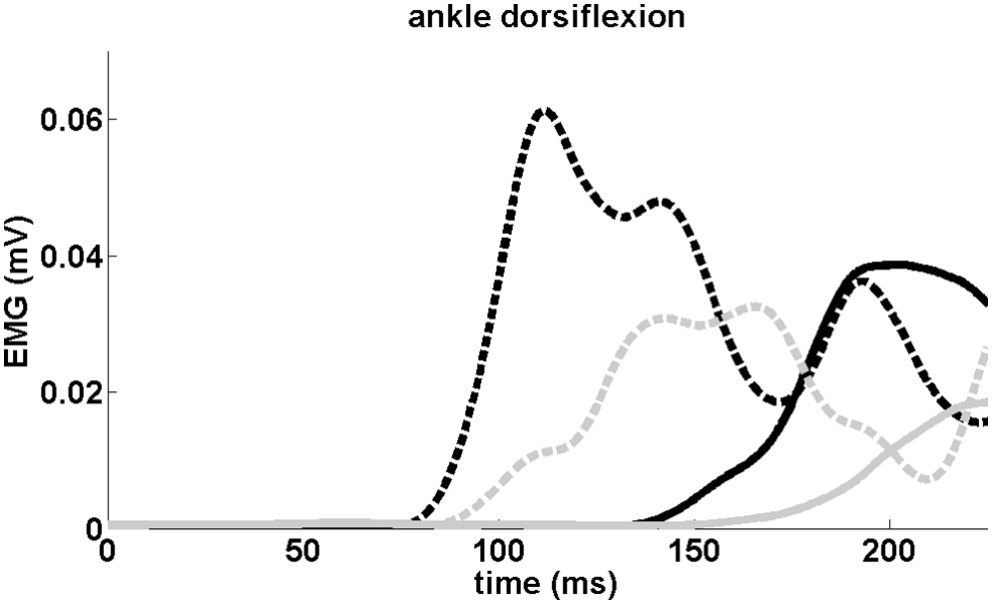
EMG signals of a representative subject from the tibialis anterior muscle during ankle dorsiflexion with the dominant leg. Grey lines represent trials after sham-tDCS, black lines after anodal-tDCS. Dotted lines represent trials with a SAS, solid lines trials without a SAS.

**Figure 2:**
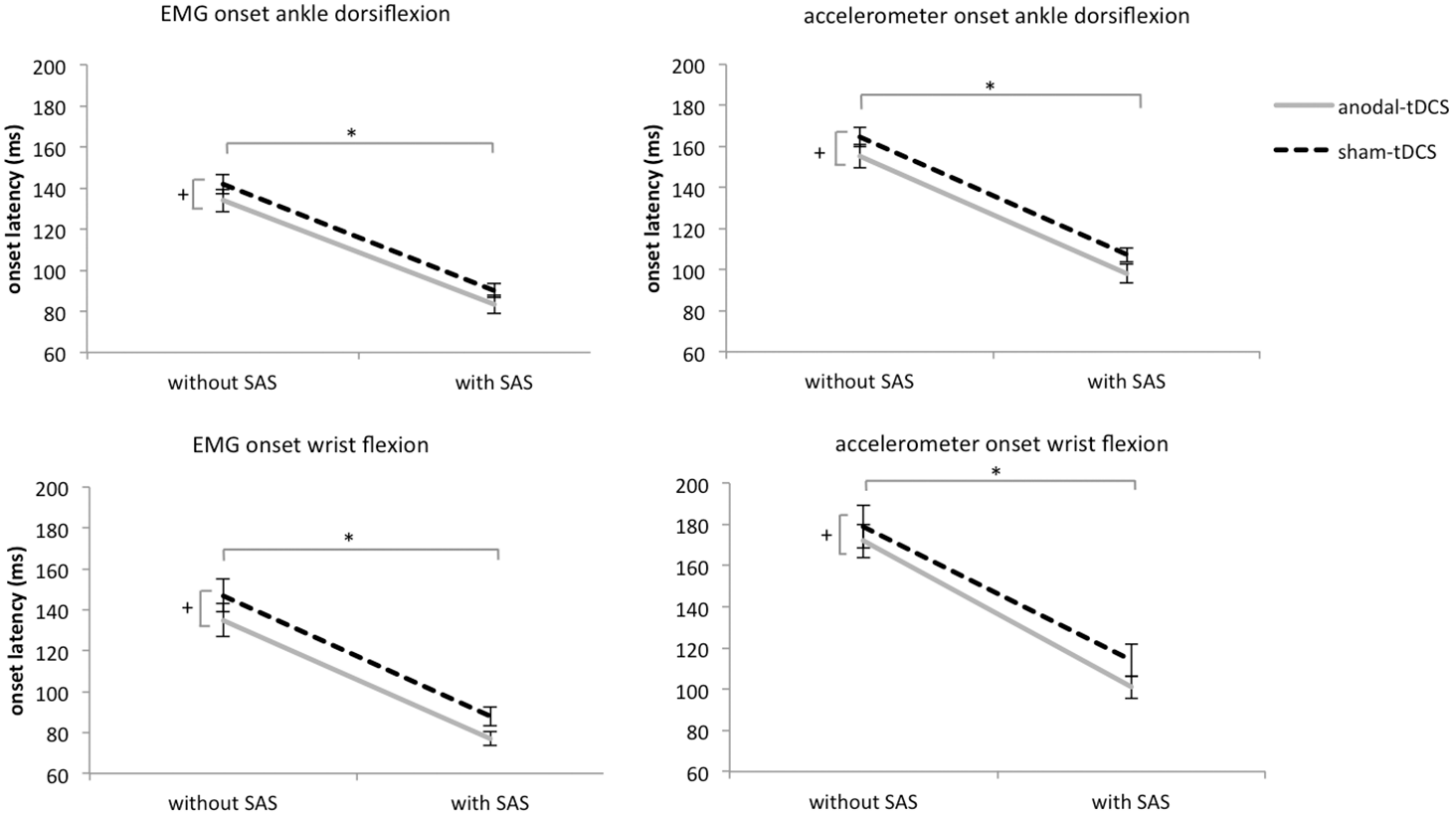
Mean onset latencies (SE) during the simple reaction time tasks involving voluntary ankle dorsiflexion and wrist flexion. *significant difference between trials with and without a SAS (main effect). +significant difference between anodal-tDCS and sham-tDCS (main effect).

The EMG traces of a representative subject during ankle dorsiflexion are shown in figure 1. Group latencies were significantly shorter after anodal-tDCS compared to sham-tDCS (7 ms shortening; *tDCS*; F_1,9_ = 13.840; p = 0.005), which effect was observed irrespective of whether or not a SAS was given (*tDCS x SAS*; F_1,9_ = 0.181, p = 0.681, see Fig. 2). A SAS significantly accelerated the onset latency of the tibialis anterior muscle, both following anodal-tDCS (51 ms acceleration) and sham-tDCS (52 ms acceleration; *SAS*; F_1,9_ = 126.642, p<0.001). Onset latencies and tDCS effects did not differ between the dominant and non-dominant leg (*leg;* F_1,9_ = 0.859 p = 0.378). The same pattern of results was obtained from the accelerometer onsets. Latencies were shorter after anodal-tDCS compared to sham-tDCS (9 ms shortening; *tDCS*; F_1,9_ = 0.327, p = 0.028, see Fig. 2), which effect was not differentially affected by the presence of a SAS (*tDCS x SAS*; F_1,9_ = 0.002, p = 0.968). Latencies were significantly accelerated by the SAS, both following anodal-tDCS (57 ms acceleration) and sham-tDCS (57 ms acceleration, *SAS*; F_1,9_ = 225.406 p<0.001). Again, we found no differences between the dominant and non-dominant leg (*leg;* F_1,9_ = 1.076, p = 0.327).

During wrist flexion, latencies of the flexor carpi radialis muscles were shorter after anodal-tDCS compared to sham-tDCS (12 ms shortening; *tDCS*; F_1,9_ = 7.306, p = 0.024, see Fig. 2), which effect was observed irrespective of whether or not a SAS was given (*tDCS x SAS*; F_1,9_ = 0.032, p = 0.868). The SAS accelerated the onset latencies, both following anodal-tDCS (58 ms acceleration) and sham-tDCS (59 ms acceleration; *SAS*; F_1,9_ = 56.416, p<0.001). This pattern was confirmed by the accelerometer data; latencies were shortened after anodal tDCS compared to sham-tDCS (10 ms shortening; *tDCS*; F_1,9_ = 7.120, p = 0.026), which effect was observed irrespective of whether or not a SAS was given (*tDCS x SAS*; F_1,9_ = 1.558, p = 0.243). A SAS accelerated the onset latencies following both anodal tDCS (71 ms acceleration) and sham tDCS (65 ms acceleration; *SAS*; F_1,9_ = 155.007, p<0.001).

### Postural responses

**Figure 3:**
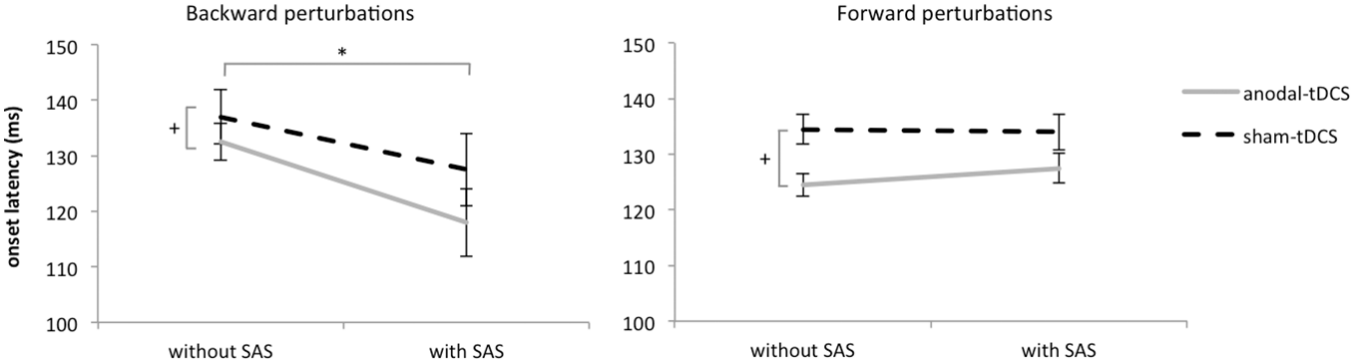
Mean onset latencies of prime movers of postural responses (SE) to backward (tibialis anterior muscle) and forward (gastrocnemius medialis muscle) perturbations. *significant difference between trials with and without a SAS (main effect). +significant difference between anodal-tDCS and sham-tDCS (main effect).

Onsets of tibialis anterior responses to backward balance perturbations were faster following anodal-tDCS compared to sham-tDCS (7 ms shortening; *tDCS*; F_1,9_ = 5.398, p = 0.045; see Fig. 3), which effect was not differentially affected by the presence of a SAS (*tDCS x SAS*; F_1,9_ = 2.408, p = 0.155). A SAS significantly accelerated response onsets to backward balance perturbations, both following anodal-tDCS (15 ms acceleration) and sham-tDCS (10 ms acceleration; *SAS*; F_1,9_ = 6.312, p = 0.033). There were no differences between the dominant and non-dominant leg (*leg*; F_1,9_ = 0.852, p = 0.380).

Gastrocnemius responses to forward perturbations were on average 10 ms faster after anodal-tDCS compared to sham-tDCS (*tDCS*; F_1,9_ = 8.484, p = 0.017, see Fig. 3). A SAS did not accelerate gastrocnemius responses to forward perturbations (*SAS*; F_1,9_ = 0.567, p = 0.471). Again, there were no differences between the dominant and non-dominant leg (*leg;* F_1,9_ = 1.289, p = 0.286).

## Discussion

In this study we aimed to establish whether transcranial direct current stimulation (tDCS) is able to facilitate subcortical motor responses in humans. We examined the effects of anodal tDCS over the non-dominant motor region on two types of motor responses that originate from subcortical structures, 1) SAS-induced wrist flexion and ankle dorsiflexion movements, and 2) postural responses to forward and backward perturbations, with and without a concurring SAS. In all tasks, responses were significantly shorter after anodal-tDCS compared to sham-tDCS, both in trials with and without a SAS. For ankle dorsiflexion as well as postural responses, the effect of tDCS did not differ between the dominant and non-dominant leg. These results support the hypothesis that tDCS facilitates not only cortical, but also subcortical structures.

### Subcortical origin of StartReact and postural responses

For the interpretation of our results, it is important to highlight the evidence for the subcortical origin of the responses studied. The origin of SAS-induced responses is a matter of an ongoing debate, but a recent study provided strong evidence that subcortical structures, in particular the reticular formation, play a key role in the StartReact effect [11]. Three hypothesis have been proposed to explain the StartReact effect. The first and prevailing hypothesis is a direct release of a subcortically stored motor program by the SAS [8], [9], conveyed by the reticulospinal tract [10], [19]. The second hypothesis proposes that the SAS could act as a subcortically mediated trigger for a cortically stored motor program, conveyed by the corticospinal tract [20], [21]. This second hypothesis is supported by the observation that the acceleration of motor responses by a SAS can be delayed by transcranial magnetic stimulation (TMS) over the motor cortex [21], [22]. Moreover, a recent study using EEG highlighted the role of cortical pre-motor areas in the preparation of SAS-induced movements [23]. Third, a SAS could act as an additional stimulus on top of the imperative stimulus, thereby increasing the energy of the sensory input, a process known as intersensory facilitation [24]. Intersensory facilitation could subsequently lead to faster sensorimotor coupling at cortical level, resulting in accelerated release of motor programs conveyed by the corticospinal tract. Importantly, SAS-induced responses are likely dissociated from startle reflexes as StartReact is often observed in the absence of standard markers of startle reflexes [25]–[30]. In a recent study we tried to unravel the hypotheses described above by applying the StartReact paradigm to patients with hereditary spastic paraplegia (HSP) [11]. HSP is a disease characterized by retrograde axonal degeneration of the corticospinal tract, while leaving the reticulospinal tract unaffected [31]. Typically, HSP in its pure form does not affect the corticospinal tracts innervating the motoneurons of the upper extremities. In our study, we compared the StartReact effect between a reaction task involving ankle dorsiflexion and a task involving wrist flexion [11]. Simple reaction times of ankle dorsiflexion were delayed in the patients with HSP compared to healthy controls, which coincided with delayed motor evoked potentials in tibialis anterior in response to supramaximal TMS. When the ankle dorsiflexion task was combined with a SAS, however, reaction times in the patients were accelerated to a larger extent than in the controls, resulting in completely normalized EMG and movement onset latencies. When the reaction time task involved voluntary wrist flexion instead of ankle dorsiflexion, no differences in onset latencies between patients and controls were recorded, irrespective of whether a SAS was applied. This pattern of results provides strong evidence for the hypothesis that a SAS accelerates reaction times by releasing a subcortically stored motor program conveyed by the reticulospinal tract.

One might argue that our study in patients with HSP provided evidence for subcortical pathways mediating SAS-induced ankle dorsiflexion responses, but that there is no direct evidence for SAS-induced wrist flexion responses originating from these structures. Yet, in people with hemiparetic stroke, a similar preservation of SAS-induced acceleration of onset latencies in the upper extremity has been demonstrated [32], [33], which suggests that these responses are also conveyed by fast subcortical pathways. Moreover, in healthy humans, StartReact responses in the upper and lower extremities exhibit the same characteristics, since they leave the muscle activation pattern unaffected and show the same degree of SAS-induced acceleration [8]. Hence, the mechanism underlying StartReact effects in the upper and lower extremities is likely the same.

There is strong evidence that, in line with StartReact responses, the reticular formation plays a key role in postural responses as well. In the present study, we investigated medium latency (automatic) postural responses that are mediated by group II or group Ib afferents. These responses have convincingly been shown not to involve transcortical pathways [12], [34], [35]. Animal studies have demonstrated that, instead, they are likely encoded by neurons in the reticular formation, which synapse onto spinal interneurons [36].

### Acceleration of automatic postural responses

Not only voluntary reaction times, but also automatic postural responses to backward balance perturbations can be accelerated by a SAS [28], [37]. In the present study, we found SAS-induced acceleration of postural responses to backward, but not to forward perturbations. These results mirror those previously reported by our group [28]. It has been hypothesized that postural responses to both forward and backward perturbations are evoked from the reticular formation, but involve different neural circuits [28] with only backward-perturbation response pathways receiving input from startle circuits. However, it remains to be investigated why the SAS-induced acceleration of postural responses is direction specific.

Although the SAS-induced acceleration of postural responses was smaller than the acceleration of voluntary movements, there is evidence that SAS-induced postural responses are consistent with a StartReact effect as well. Previous studies have demonstrated that a SAS can trigger a voluntary movement at similarly short onset latencies when applied in the absence of the imperative signal [18], [38], [39]. This characteristic of StartReact responses has proved to be applicable to postural responses as well. Two studies have shown that postural responses can be triggered by a SAS in the absence of a balance perturbation [28], [37] with similar latencies to those in the presence of a perturbation [28]. Because of this observation, it is unlikely that the SAS-induced shortening of postural response latencies is due to intersensory facilitation. Furthermore, the observation of unidirectional SAS-induced acceleration of postural responses is also not consistent with intersensory facilitation, as this mechanism would likely accelerate responses to both forward and backward perturbations.

### Subcortical structures can be facilitated by tDCS

This study provides evidence for tDCS-induced subcortical facilitation in humans. These findings are in agreement with the recently reported facilitation of reticulospinal and rubrospinal motor neurons by tDCS in anaesthetized cats [6]. Similar has also been reported in rats [40], albeit evoked by cathodal-stimulation. A previous observation already hinted at tDCS-induced subcortical facilitation in humans, but direct evidence was lacking. It was reported that during and following tDCS there was an increase in regional cerebral blood flow in subcortical structures, including the red nucleus and the mesencephalic and pontine reticular nuclei [7]. This observation may point at an effect of tDCS at the subcortical level, but its functional significance could not be established. The present results demonstrate that tDCS application indeed changed the excitability of subcortical structures, leading to faster response onsets.

The facilitation of subcortical structures by tDCS may be explained by two mechanisms that are not mutually exclusive. First, the subcortical facilitation may have resulted from a change in the cortico-reticular drive. Second, the applied current may have directly changed the excitability of subcortical structures. The latter hypothesis is supported by a modeling study on the spread of current during tDCS application using the same electrode configuration as in this study, which demonstrated the potential for direct subcortical effects [41]. Both mechanisms were found when tDCS was applied to anaesthetized cats in the study of Bolzoni *et al.* [6].

Alternatively, one might argue that the present results may be explained by an increased arousal or general attention caused by tDCS, which was not present during the sham condition. An increase in arousal or attention could have affected both cortical and subcortical pathways. Indeed, it has shown that tDCS can improve attention and thereby reduces reaction times, likely via facilitation of cortical structures [42]. The effect of attention or general arousal on SAS-induced reaction times has not been investigated, which leaves the possibility of increased general arousal underlying the observed reduction in SAS-induced onset latencies following tDCS. However, this explanation does not seem to hold true for the observed acceleration of postural responses, as there are several studies that suggest that onset latencies of these responses are not influenced by attention or arousal. For instance, responses onsets do not change when attention has to be divided between a postural and a concurrent cognitive task [43], [44]. Moreover, in a study that evaluated automatic postural responses to external perturbations in participants while standing in a high postural threat condition, response onsets in the lower extremities were not influenced by anxiety [45]. Hence, it seems unlikely that the acceleration of subcortical motor responses by tDCS as found in the present study can solely be attributed to increased general arousal or attention.

### Role of the reticular formation in the StartReact effect and postural responses

The present results raise the question which subcortical structures can be facilitated by tDCS. In the study of Bolzoni et al., tDCS application in anaesthetized cats yielded direct and indirect facilitation of reticulospinal motor neurons [6]. There are several arguments why this may have been the mechanism underlying the present results as well. There is compelling evidence that the pontomedullary reticular formation (pmRF) is critically involved in generating the automatic postural responses to external balance perturbations [36], [46] as well as in the StartReact effect [11]. Studies in monkeys and cats have indentified the pmRF as one of the subcortical structures that subserves motor preparation [47], [48]. As the pmRF is also a key structure in the startle reflex circuitry [49], [50], it presumably plays a pivotal role in the release of pre-prepared motor programs, resulting in the StartReact effect. Hence, the acceleration of fast SAS-induced ankle and wrist movements and of automatic postural responses following tDCS application over the sensorimotor cortex most likely results from facilitation of the reticular formation. In contrast, responses during reaction time tasks without a SAS most likely originate from the cortex. The tDCS-induced acceleration of ankle and wrist movements during trials without a SAS therefore point to facilitation of cortical structures, which is in line with previous work [51], [52].

### Bilateral effects of tDCS

Although the anodal electrode was always positioned over the non-dominant motor region, we found no differences in the effects of tDCS between the dominant and non-dominant leg, both for cortically and subcortically organized responses. One reason may be that the applied current was rather large, which may have resulted in a significant spread of current across the brain also affecting subcortical structures, including cortico-reticular pathways and the brainstem reticular formation. The bilateral effects of tDCS on subcortical structures may thus be explained by a direct (bilateral) effect of tDCS on the reticular formation, or by a change in the cortico-reticular drive. Although cortico-reticular projections are predominantly ipsilateral in humans [14], [53], contralateral projections have also been identified [54], [55].

Responses during reaction time tasks without a SAS, which are mediated by cortical structures, were also bilaterally accelerated by tDCS. This suggests that the direct cortical effects of tDCS were not strictly lateralized either, but the underlying mechanism remains to be investigated. The bilateral effects may also be due to ipsilateral connectivity to lower limb motor neurons [56]. Alternatively, we cannot rule out that bilateral cortical effects resulted from increased arousal evoked by tDCS.

### Future studies

The present results have implications for future studies investigating the effects of tDCS on cortically mediated responses. As our study demonstrates that the common application of tDCS over the sensorimotor cortex also yields effects on a subcortical level, the possibility of such effects interacting with the cortical effects of interest should be considered. In addition, future studies may investigate how the subcortical effects of tDCS can be enhanced. Bolzoni *et al.* reported that the facilitation of subcortical structures in anaesthetized cats is enhanced by repeated application of tDCS [6]. We hypothesize that this may also be the case in humans, but this needs to be proven. Furthermore, a modeling study has suggested that the facilitation of ventrally located subcortical structures (i.e., the brainstem) might be larger with the reference electrode placed in contact with the neck muscles (extracephalic position) compared to a supraorbital position [41]. Our paradigm might be useful to study this hypothesis. However, the effects of the extracephalic positioning of the reference electrode should be closely monitored, as a case study reported on disturbed breathing, speech arrest and psychosis after brainstem stimulation [57], [58].

### Application in clinical practice

As subcortical structures, in particular the reticular formation, are involved in motor preparation they could play a compensatory role in the recovery after corticospinal lesions [59]. A recent study in monkeys suggested that the reticulospinal tract is indeed responsible for some functional recovery after acute corticospinal lesions, such as stroke [60]. It has also been suggested that a similar compensatory mechanism may be at work in patients with hereditary spastic paraplegia [11]. Compensation by the reticular formation requires strengthening of the output, not the growth of new neural connections [59]. The application of tDCS may, therefore, be useful to increase the activation of reticulospinal motoneurons or result in a stronger reticulospinal output, both of which could be beneficial for motor recovery and rehabilitation [6]. Interestingly, a recent study in patients with leukoaraoisis (hyperintensities in the subcortical white matter) showed that balance performance improved in response to a combined session of physical training and tDCS over the midline motor and premotor areas, but not following physical training alone [61]. In light of the present results, these improvements may have resulted from tDCS-induced reticulospinal facilitation.
